# Silent wounds: an epidemiological analysis of self-inflicted injuries among youths in Brazil (2013-2023)

**DOI:** 10.1590/0102-311XEN062525

**Published:** 2026-03-02

**Authors:** Gabriela Garcia de Carvalho Laguna, Ana Luiza Ferreira Gusmão, Ana Beatriz Ferreira Gusmão, Jéssica Sabrina Gonçalves Fernandes, Yago Soares Fonseca, Katiene Menezes Rodrigues de Azevedo

**Affiliations:** 1 Instituto Multidisciplinar em Saúde, Universidade Federal da Bahia, Vitória da Conquista, Brasil.; 2 Universidade Federal do Sul da Bahia, Itabuna, Brasil.

**Keywords:** Self-Injurious Behavior, Suicide, Child, Adolescent, Comportamento Autodestrutivo, Suicídio, Criança, Adolescente, Conducta Autodestructiva, Suicidio, Niño, Adolescente

## Abstract

This study aimed to describe the epidemiological profile of self-inflicted injuries among children and adolescents in Brazil over the past decade (2013-2023). This ecological study had a nationwide coverage and was based on data from the Brazilian Health Informatics Department (DATASUS). Descriptive and inferential statistical analysis were applied (t-test, ANOVA, Tukey, and Friedman), with normality assessment (Shapiro-Wilk), using Jamovi software. From 2013 to 2023, 18,382 hospitalizations and 261 deaths due to self-inflicted injuries were recorded among children and adolescents in Brazil, with a total hospital cost of approximately BRL 10 million. The Southeast accounted for the highest number of hospitalizations (55.45%) and deaths (60.1%), while the North reported the lowest figures. The most affected age group was 15-19 years. Hospitalizations were more frequent among females, whereas deaths predominated among males, with a significant impact on the Black population. During the study period, hospitalizations increased by 44.28% and deaths by 26.31%, with the highest hospital costs occurring in 2022 and 2023. These findings reveal significant regional and demographic disparities and underscore the need for targeted prevention strategies and specific public health policies.

## Abstract (pt)

Esse estudo buscou descrever a situação epidemiológica das lesões autoprovocadas em crianças e adolescentes no Brasil nos últimos dez anos (2013-2023). Este estudo ecológico com abrangência nacional foi baseado em dados do Departamento de Informação e Informática do SUS (DATASUS). Estatísticas descritivas e inferenciais (teste t, ANOVA, Tukey e Friedman) e verificação de normalidade (Shapiro-Wilk) foram aplicadas no software Jamovi. O Brasil registrou 18.382 internações e 261 óbitos por lesões autoprovocadas entre crianças e adolescentes de 2013 a 2023, acarretando um custo hospitalar total de cerca de 10 milhões de reais. A Região Sudeste foi responsável pelo maior número de internações (55,45%) e óbitos (60,1%), enquanto a Região Norte registrou os menores números. A faixa etária entre 15 e 19 anos foi a mais acometida. As internações foram mais frequentes entre as mulheres, enquanto os óbitos predominaram entre os homens, com impacto significativo na população negra. Houve um aumento de 44,28% nas internações e um aumento de 26,31% nos óbitos no período, com os maiores custos hospitalares ocorrendo em 2022 e 2023. A análise destaca disparidades regionais e demográficas significativas, ressaltando a necessidade de estratégias de prevenção direcionadas e políticas públicas específicas.

## Abstract (es)

El objetivo era describir la situación epidemiológica de las lesiones autoinfligidas en niños y adolescentes en Brasil durante los últimos 10 años (2013-2023). Se trata de un estudio ecológico con cobertura nacional, basado en datos del Departamento de Informática del SUS (DATASUS). Se aplicaron estadísticas descriptivas e inferenciales (t-test, ANOVA, Tukey y Friedman), con verificación de normalidad (Shapiro-Wilk), utilizando el *software* Jamovi. Entre 2013 y 2023, se registraron 18.382 hospitalizaciones y 261 muertes por lesiones autoinfligidas entre niños y adolescentes en Brasil, con un coste hospitalario total de aproximadamente 10 millones de reales. La región Sudeste registró el mayor número de hospitalizaciones (55,45 %) y muertes (60,1 %), mientras que la región Norte registró las cifras más bajas. El grupo de edad más afectado fue el de 15 a 19 años. Las hospitalizaciones fueron más frecuentes entre las mujeres, mientras que las muertes predominaron entre los hombres, con un impacto significativo en la población negra. Se produjo un aumento del 44,28% en las hospitalizaciones y del 26,31% en las muertes durante ese periodo, y los costes hospitalarios más elevados se registraron en 2022 y 2023. El análisis pone de relieve importantes disparidades regionales y demográficas, lo que subraya la necesidad de estrategias de prevención específicas y políticas públicas concretas.

## Introduction

The concept of self-inflicted injury encompasses a broad spectrum of behaviors in which an individual intentionally harms themselves, with or without suicidal intent ^1^ ^,^ ^2^. Non-suicidal self-injury refers to deliberate self-harm without the intention to die, often serving as a coping mechanism to regulate negative emotions and obtain emotional relief ^3^ ^,^ ^4^. In contrast, self-inflicted injuries with suicidal intent - some resulting in unsuccessful suicide attempts and others without an explicitly expressed death wish - are associated with a higher hospital mortality. The distinction between non-suicidal self-injury and suicidal behavior remains complex, as these phenomena frequently overlap and share common risk factors ^5^. Additionally, cultural, psychological, and methodological differences across studies contribute to variations in how self-injury is conceptualized and classified, highlighting the challenge of establishing clear distinctions in both clinical and epidemiological contexts ^5^ ^,^ ^6^.

During adolescence, various risk factors contribute to mental distress, including exposure to adversities, parental psychopathology, recurring episodes of bullying or threats, excessive time spent on social media, as well as hormonal changes and developmental processes related to the formation of personal identity and individuality ^7^ ^,^ ^8^ ^,^ ^9^. As a result, self-injury is often used as a coping strategy for emotional regulation during this stage ^10^ ^,^ ^11^. In this context, it is important to address bullying and its variant, cyberbullying, which involve repetitive verbal, physical, social, or electronic aggression directed at individuals based on characteristics that distinguish them from the group. This form of oppression is not exclusive to a specific age group but is significantly more prevalent among children and adolescents. Therefore, increased attention to this population is essential to prevent self-injurious behaviors or suicide attempts resulting from such experiences ^11^.

Brazil, mirroring global trends, has also experienced rising rates of self-injurious behavior. Reported cases in the country increased from 14,940 in 2011 to 89,272 in 2018, an increase of nearly 500% ^12^ - despite underreporting, as this is a sensitive issue. Several studies have examined self-injury among adolescents, often focusing on specific populations. Research conducted in Mato Grosso State described a profile characterized by a higher prevalence among females aged 15-19 years, with poisoning as the most common method ^13^. Similarly, a study in Palmas (Tocantins State), a capital city in the Amazon region, reported self-inflicted violence among adolescents, predominantly in females aged 15-19 years, with poisoning and sharp objects as the main mechanisms ^14^. A study using data from the Brazilian Information System for Notifiable Diseases (SINAN, acronym in Portuguese) analyzed intentional self-inflicted injuries among adolescents in the school environment ^15^. Approximately a decade ago, a nationwide analysis of hospitalizations for self-inflicted injuries among adolescents also identified a higher prevalence among females, along with a growing trend in the Southeast ^15^ ^,^ ^16^.

Regarding costs of hospitalizations for self-harm, a study conducted in Australia estimated an annual burden of AUD 55 million to the health system, with an average cost per episode of USD 4,649; hospitalizations for self-harm were 21 times more frequent than deaths by suicide ^17^. Other studies not limited to children and adolescents have also found significant economic impacts. In England, the annual cost of self-harm management was GBP 162 million, with an average cost of GBP 809 per episode ^18^. In Brazil, a recent study analyzed costs associated with self-inflicted violence between 2010 and 2020, estimating a total expenditure exceeding BRL 30.8 million, with an average cost of BRL 949 per hospitalization, particularly in the South and Southeast ^19^. Although these data help delineate the scope of the issue, they still lack updated and comprehensive national estimates of costs related to self-inflicted injuries. Therefore, accurate cost estimates are essential to support healthcare management decision-making and the development of prevention strategies, especially among the pediatric population.

These studies demonstrate the growing importance and increasing prevalence of this phenomenon. However, no studies published within the past decade were identified that encompassed hospitalizations, deaths, and costs among children and adolescents, capable of more accurately reflecting the current reality and informing future policy-making. Although some investigations provide relevant information on self-injury among children and adolescents, they are often limited to specific regions, such as the Amazon or Mato Grosso State, and therefore do not capture the broader Brazilian context ^14^ ^,^ ^15^. Conversely, studies with wider national coverage typically include data only up to 2016 or 2018 ^16^ ^,^ ^20^. Since then, major events have occurred, such as the COVID-19 pandemic, underscoring the need to assess whether epidemiological patterns and the economic burden of self-inflicted injuries in Brazil have changed.

Thus, more recent studies are needed to expand the available data by providing a socioeconomic characterization and detailed patterns of self-inflicted injuries during both childhood and adolescence. Such analyses should include not only hospitalization rates but also deaths and healthcare-related costs. This study aims to describe the evolution of the epidemiological profile of self-harm among children and adolescents in Brazil from 2013 to 2023, highlighting regional and demographic trends and variations.

## Method

This ecological study with national coverage used demographic data from the Brazilian Institute of Geography and Statistics (IBGE, acronym in Portuguese) ^21^ and health data from the Brazilian Hospital Information System (SIH, acronym in Portuguese), managed by the Brazilian Health Informatics Department (DATASUS, acronym in Portuguese) ^22^. Both data sources are open access and contain no personally identifiable information. Therefore, approval from a Research Ethics Committee was not required, as the study relied exclusively on secondary data. The study was conducted in accordance with the Strengthening the Reporting of Observational Studies in Epidemiology (STROBE) guidelines ^23^ and was completed in July 2024.

The characterization of the Brazilian population was based on data from the 2022 Census, as the previous 2010 Census did not fall within the study period. Information on the total population was collected according to gender, age group, and region of residence in Brazil. Data on hospitalizations, deaths, mortality rates, and hospital care costs related to intentionally self-inflicted injuries (codes X60-X84 from the International Classification of Diseases, 10th revision - ICD-10) were analyzed and stratified by age group (5-19 years), gender, race, and Brazilian region, covering the period from January 2013 to December 2023. Given the methodological challenges in studying self-inflicted injuries, these classifications help minimize misinterpretations, particularly among readers unfamiliar with the subject.

To ensure consistency, racial classification in the text and figures follows the IBGE categories, with “Black” referring to the combined group of individuals who self-identify as Black or Mixed-race. Racial composition is described accordingly to enhance clarity.

Quantitative data were initially analyzed using descriptive statistics in absolute numbers and percentages. The software Jamovi version 2.5.6 (https://www.jamovi.org) was employed for statistical analysis. For repeated-measures analyses, normality was evaluated using the Shapiro-Wilk test (p > 0.001). To assess differences in the number of hospitalizations and deaths across Brazilian regions and age groups, a repeated-measures analysis of variance (ANOVA) was conducted, followed by Tukey’s post-hoc test. ANOVA was used to compare means of dependent variables (number of hospitalizations and deaths) across the fixed factors of region and age group, enabling the identification of significant variations across these categories (p < 0.05). The use of a repeated-measures design was appropriate since the same population groups were evaluated across multiple regional and age categories. After identifying significant differences, Tukey’s post-hoc test was applied to determine specific comparisons showing statistically significant differences (p < 0.01). Data distribution was presented using graphs.

### General characterization of the study location

Brazil covers an area of 8,510,417.77km^2^ and is divided into five macroregions: North, Northeast, Central-West, Southeast, and South. The total population is approximately 203 million inhabitants, distributed as follows: 41.78% in the Southeast, 28.3% in the Northeast, 14.74% in the South, 8.55% in the North, and 6.63% in the Central-West. Regarding gender and race, approximately 51.5% of the population is female, and 55.5% identify as Black (45.3% as Mixed-race and 10.2% Black), 43.5% as White, 0.6% as Indigenous, and 0.4% as Asian.

Given that this study aims to explore regional differences, a broader contextualization of Brazil’s socioeconomic landscape was included, extending beyond a simple description of the five macroregions. This approach strengthens the international relevance of the study and adds depth to the discussion. Additionally, to improve the classification of self-inflicted injuries, the study proposes refining codes X60-X84 to specify the methods used, regardless of the injury stage.

The age structure of the country is predominantly composed of adults and older adults, although children and adolescents still represent a substantial proportion of the population. Specifically, 6.25% are under five years of age, 6.77% are aged 5-9 years, 6.73% are 10-14 years, and 7.08% are 15-19 years. Adults aged 20-29 account for 15.22%, those aged 30-39 for 15.58%, and those aged 40-49 for 14.62%. Individuals aged 50-59 comprise 11.89%, followed by 8.77% aged 60-69, 4.77% aged 70-79, and 2.24% aged 80 years or older. Regarding data access and methodology, the study explicitly details the calculation of prevalence (including new and existing cases) rather than incidence (new cases only), aligning with the study period, age groups, and regional analyses. The study also examines the financial burden associated with hospitalizations due to self-inflicted injuries. Costs were estimated using hospital expense records available from DATASUS, based on values reported for hospital admissions. Both direct hospitalization costs, as recorded for inpatient procedures, and other medical expenses incurred during the hospital stay, as documented in the system’s databases, were included.

## Results

In Brazil, between January 2013 and December 2023, 18,382 hospitalizations and 261 deaths due to intentional self-harm were recorded among children and adolescents aged 5-19 years, highlighting the seriousness of the issue and its implications for youth mental health. In addition to the high human burden, these events generated a significant financial impact, with healthcare expenditures totaling approximately BRL 10 million over the study period. These figures are presented as absolute numbers and were not adjusted for the population size of each region or age group.

**Figure 1:**
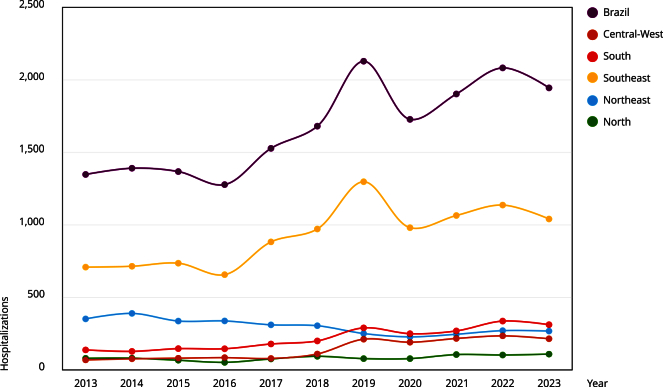
Number of hospitalizations for intentionally self-inflicted injuries in Brazilian macroregions by year.

Source: Brazilian Hospital Information System (SIH) ^22^.

Statistical analysis using ANOVA revealed significant differences in the number of hospitalizations and deaths across Brazilian regions (p < 0.01). Tukey’s post-hoc test indicated that the Southeast had significantly higher numbers of hospitalizations and deaths compared to the South and Central-West, while the North had significantly fewer cases than all other regions. No differences were observed over time, indicating stability in annual variation. The Southeast recorded the highest number of hospitalizations and deaths, accounting for 10,194 hospitalizations (55.45%) and 157 deaths (60.1%). The Northeast ranked second in cumulative hospitalizations, representing 17.93% of the total during the study period; however, the observed downward trend in hospitalizations was not statistically significant. Nationwide, a 44.28% increase in hospitalizations was observed when comparing values from 2013 to 2023. Figure 1 shows the annual number of hospitalizations due to intentionally self-inflicted injuries across Brazilian regions within the Brazilian Unified National Health System (SUS, acronym in Portuguese).

**Figure 2:**
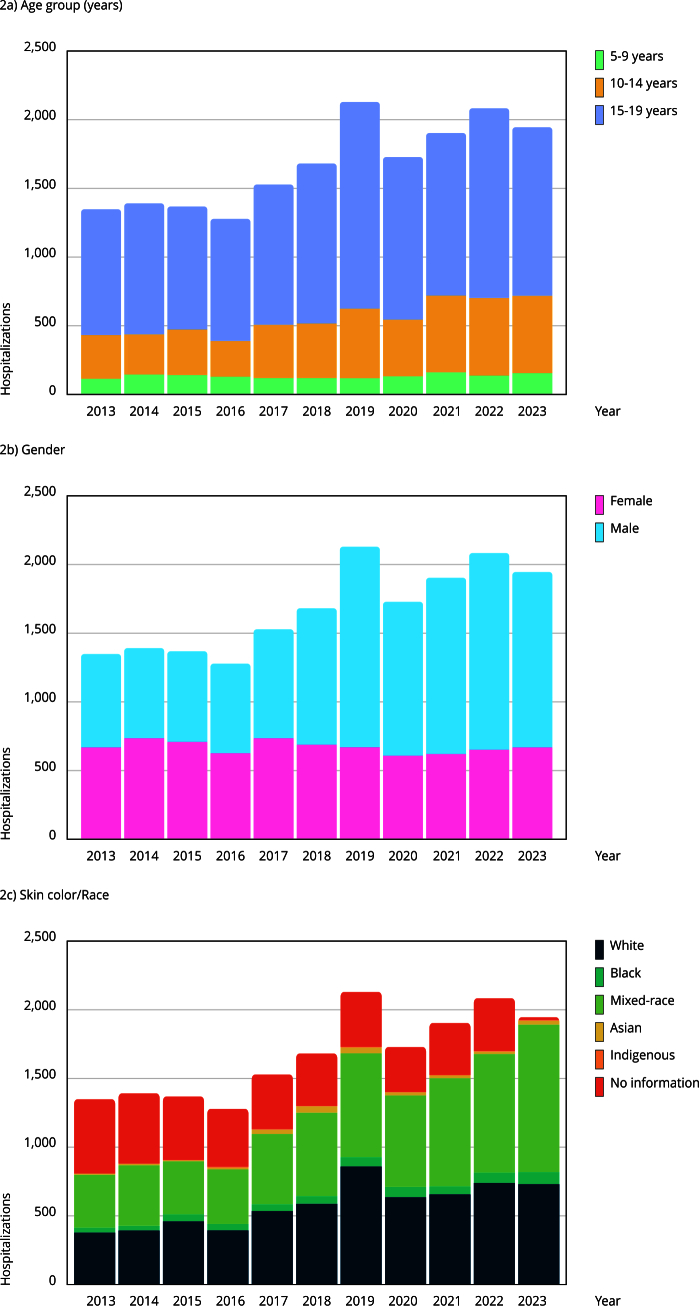
Number of hospitalizations for intentionally self-inflicted injuries by skin color/race, gender, and age group by year.

Source: Brazilian Hospital Information System (SIH) ^22^.

Analysis by age group showed significant differences in the number of hospitalizations across regions (p < 0.001). Tukey’s post-hoc test revealed that children aged 5-9 years had significantly fewer hospitalizations compared to other age groups. Adolescents aged 15-19 years were the most affected, representing 67.03% of all hospitalizations (n = 12,322). Figure 2 shows the distribution of hospitalizations by Brazilian region between 2013 and 2023.

Regarding skin color/race and gender, the highest numbers of hospitalizations and deaths were observed among Black individuals (combined Black and Mixed-race), totaling 7,509 hospitalizations (40.84%), followed by White individuals, with 6,371 hospitalizations (34.65%). Hospitalizations occurred predominantly among females, totaling 10,995 cases (59.81%).

The analysis of deaths showed that the highest mortality occurred in the 15-19 year age group (p < 0.001), with 211 deaths (80.84%). Mortality was also higher among Black individuals, totaling 109 deaths (41.76%). In terms of gender, deaths were more frequent among males, with 154 deaths (59%). Over the study period, the number of deaths increased by 26.31%, rising from 19 in 2013 to 24 in 2023, with the highest peak in 2020 (30 cases), followed by 2019 (27 deaths). Figure 2 shows the distribution of hospitalizations by skin color/race, gender, and age group from 2013 to 2023.

Regarding costs, a distribution similar to that of hospitalizations was observed. The Southeast accounted for 57.47% of total costs (BRL 5,753,468.6), while adolescents aged 15-19 represented 72.84% of total spending during the study period (BRL 7,292,203). Costs were also highest among Black individuals (combined Black and Mixed-race), totaling BRL 4,201,784.67 (41.97% of the total), and among females, with BRL 5,246,809.5 (52.41% of national spending).

## Discussion

This study identified a growing prevalence of self-inflicted injuries during childhood and adolescence in recent years. Developing preventive strategies is crucial, considering the risk of child suicide ^20^. The results align with a Brazilian study reporting increases in all forms of childhood violence, particularly self-inflicted injuries ^24^. It is essential to acknowledge the possibility of underreporting, a recognized limitation in the epidemiology of self-inflicted injuries across all age groups, which is particularly acute in children and adolescents ^25^. Underreporting can significantly influence incidence findings due to variability and incompleteness of reporting systems. This issue has serious implications for the healthcare system: as long as underreporting persists, children and adolescents in distress may remain largely invisible to public authorities, hindering accurate assessment of population health and the effective development of targeted public policies.

In analyzing these data, it is imperative to address methodological limitations inherent to the use of the SIH. Because the SIH primarily captures data from SUS and not the entire national healthcare system, our findings must be interpreted within this context ^26^. Consequently, cases involving children and adolescents who access private or supplementary health services are not represented, which may lead to underestimation of self-inflicted injury rates among higher-income groups. This limitation highlights the structural fragmentation of health information systems in Brazil and reinforces the need for integrated data encompassing both public and private networks to provide a more comprehensive view of the national burden. As a result, the data may disproportionally reflect populations that are socioeconomically vulnerable and rely exclusively on SUS. This supports the hypothesis that the burden of self-inflicted injuries is concentrated among Brazil’s most vulnerable groups, including lower-income individuals and those identifying as Black or Mixed-race - a finding consistent with existing literature on structural inequality and mental health access ^27^ ^,^ ^28^. This framework is essential for interpreting subsequent findings on regional and racial/ethnic disparities.

The study revealed regional differences in hospitalization rates for self-inflicted injuries in Brazil. This contrasts with a prior epidemiological study, which reported increases in self-inflicted injuries in the Southeast and Central-West from 2013 onwards ^24^. In our data, however, the upward trend in hospitalizations was most pronounced in the Southeast, South, and Northeast, followed by the Central-West. Interpreting these regional disparities requires considering multiple factors: population density, greater access to technological resources for case reporting in more developed regions, and variability in SUS coverage and quality across the country, as previously discussed. Socioeconomic and ethnic distributions across regions are also essential, as vulnerable populations, who predominantly rely on public health services, are unevenly distributed, contributing to the observed geographic concentration of the burden. Therefore, local public policies for child and adolescent protection must provide targeted socioeconomic and psychological support, addressing regional, social, and structural inequalities.

Consistent with national and international research highlighting the disproportionate impact of self-harm on vulnerable groups, this study identified the highest numbers of hospitalizations and deaths among Black individuals (encompassing both Black and Mixed-race categories) ^27^. Socioeconomic issues, parental unemployment, and chronic lack of access to basic services are directly associated with mental distress in this pediatric population ^28^. These findings reinforce the need for equity-focused public policies that provide targeted psychosocial support, promote healthy mental development, and mitigate the compounding effects of systemic disadvantage. Furthermore, it is essential to highlight the methodological challenge posed by the high percentage of individuals with undefined race/skin color in records. This data gap hinders the precise assessment of self-inflicted injuries across ethnic groups, undermining efforts to address racial disparities. Accurate recording of race/skin color in clinical records and careful transmission to reporting systems are therefore critical, ensuring that such information can be effectively utilized in epidemiological studies, which serve as the foundation for informing interventions aimed at addressing this issue.

The highest number of deaths occurred among males, consistent with findings from previous research ^29^ ^,^ ^30^. A study on childhood suicide identified a higher prevalence among older children and adolescents, with hanging and firearms being the most common methods of self-harm ^24^. This underscores the need for strategies to restrict firearm access for children and adolescents and to enhance family protection and support during this life stage. This study also found that hospitalizations and deaths predominantly affected adolescents aged 15-19 years, representing over 80% of the studied population. This pattern aligns with global studies, as this age group is more susceptible to anxiety, depression, and other mental health issues ^24^ ^,^ ^31^.

It is also critical to address methodological challenges associated with classifying self-inflicted injuries, particularly when utilizing administrative data systems such as the SIH, which rely on the ICD-10 (codes X60-X84). These codes are designed to capture the intent of the injury (intentional self-harm), which is crucial for epidemiological surveillance. However, they present limitations in specifying the method used (e.g., hanging, poisoning, or cutting) and in distinguishing between non-suicidal self-injury and a high-lethality suicide attempt. The broad nature of the X60-X84 classification can challenge readers unfamiliar with the subject and, more importantly, hampers the development of targeted prevention strategies ^32^. To overcome this limitation, this study reinforces the need to refine the X60-X84 codes in clinical records and reporting systems to specify the methods used, regardless of the perceived injury stage. Such detailed data are foundational to accurately assess risk, restrict access to lethal means (such as firearms and chemicals), and design evidence-based public health interventions tailored to the specific behaviors and risks observed in the pediatric population.

A systematic analysis from the 2019 *Global Burden of Disease Study* highlighted that adolescent mortality in Brazil remains persistently high ^33^. Similarly, our study describes higher mortality due to self-inflicted injuries among older children and adolescents. This underscores challenges in formulating policies to address global health risks within the pediatric population, particularly among adolescents. Despite acknowledgment of increasing mortality rates, funding for this age group remains insufficient. Therefore, it is essential to reduce inequalities, promote access to mental health services, and improve data quality. These data should also inform health policies, including investments in evidence-based interventions and measures to restrict access to firearms and chemicals commonly used for self-harm.

A meta-analysis identified that self-harm is often a negative coping strategy to relieve pressure or tension resulting from adverse interpersonal events ^34^. In this regard, healthcare and education professionals are encouraged to monitor psychosomatic complaints in children or adolescents, regularly inquire about bullying or other challenging situations, and assess emotional functioning and the need for professional support before self-harm emerges as a coping mechanism. Nevertheless, it is relevant to highlight that the true prevalence of self-inflicted injuries remains unknown, as these behaviors frequently occur in private, leaving silent wounds that are difficult for others to detect.

An analysis of temporal trends revealed a pattern strongly related to the COVID-19 pandemic. While 2020 coincided with the implementation of social distancing and isolation measures, an initial drop in reported hospitalizations for self-inflicted injuries was observed, followed by a marked increase in 2021 and 2022. This reduction in 2020 should be interpreted with caution, as it likely reflects severe underreporting, as has been the case with other diseases in the country ^35^. During the peak of the pandemic, public health services were overwhelmed with respiratory illnesses, and access to routine care - along with child protection surveillance mediated by schools and other networks - was severely limited. Concurrently, the highest number of deaths from self-inflicted injuries in the pediatric population occurred in 2020. This discrepancy (fewer reported cases but increased mortality) suggests that the underlying psychosocial impact of the pandemic - marked by social isolation, reduced access to protective factors (such as school and leisure activities), socioeconomic difficulties, and interpersonal conflicts - may have led to more severe episodes or suicide attempts involving highly lethal methods. Evidence also indicates that increased social media use, especially among adolescents, was associated with greater mental distress during the pandemic, while family support and access to academic activities were crucial protective factors ^28^ ^,^ ^29^ ^,^ ^36^. Therefore, despite a temporary decrease in reported cases, the intensity of distress and lethality of injuries appear to have peaked in 2020, highlighting the urgent need for specific mental health interventions during periods of major social crisis.

The highest costs associated with self-inflicted injuries in children and adolescents were recorded in 2022 and 2023, particularly among Black individuals and females. A Brazilian study conducted during the pandemic identified higher levels of anxiety among female children and adolescents ^37^. Over the studied period, a 67.52% increase in hospital costs was observed regarding self-inflicted injuries. These data suggest that the social and economic repercussions of this issue are growing in Brazil, highlighting the need for targeted public policies. These costs could be reduced, and quality of life, especially regarding mental health, could be improved with the implementation of self-harm prevention strategies and life-affirming interventions ^38^.

Future research should explore the underlying causes of self-inflicted injuries and identify prevention strategies targeted at the most affected regions and age groups. The implementation of public policies, early intervention programs, and psychological support is essential to reduce the prevalence, injury severity, and associated costs. Initiatives from multiple actors, including nongovernmental organizations, schools, and private institutions, are also important in addressing this issue. Parental support and a healthy parent-child bond emerge as clear coping strategies for self-harm. Another alternative involves integration into a peer group in which self-injurious behaviors are shared as lived experiences among children and adolescents, addressing their consequences and ways for this population to receive support.

### Limitations

A limitation of this study is the use of the SIH to estimate hospitalizations, as it excludes data from non-contracted private hospitals, limiting comparability with SINAN data. Additionally, underreporting of self-inflicted injuries, as not all cases are recorded in the health system, may hinder a comprehensive understanding of the issue and the development of effective intervention policies.

## Conclusion

This study analyzed epidemiological trends of self-inflicted injuries among Brazilian children and adolescents (2013-2023), focusing on hospitalizations, mortality, and associated costs. Results show a progressive increase in hospitalizations, particularly in the Southeast, which recorded the highest absolute numbers of cases and deaths. When adjusted for population distribution, other regions also presented notable proportional burdens. The most affected group was adolescents aged 15-19 years, with higher rates among Black individuals and females. Hospital-related expenditures increased by 67.52%, peaking in 2022. These findings demonstrate persistent regional and demographic disparities, emphasizing the need to consider population proportionality in future analyses. Targeted prevention strategies, strengthened public policies, and expanded access to psychological support are necessary to address these inequalities and reduce the impact of self-inflicted injuries on the healthcare system.

## Data Availability

The sources of information used in the study are indicated in the body of the article.
